# Multiple analysis of the nutritional status of children and adolescents in Western China

**DOI:** 10.1017/S0007114526106400

**Published:** 2026-05-28

**Authors:** Yan Sun, Lianlong Yu, Hua Guo, Yiya Liu

**Affiliations:** 1 Guizhou Center for Disease Control and Prevention, Guizhou, People’s Republic of China; 2 Shandong Provincial Center for Disease Control and Prevention, Shandong, People’s Republic of China

**Keywords:** Children and adolescents, Nutritional status, Malnutrition, Overnutrition

## Abstract

Nutrition is crucial for the growth of children and adolescents. This study investigated multiple nutritional problems and influencing factors among 2423 students aged 6–17 years in Guizhou Province, using questionnaire surveys, physical examinations and blood tests. Multifactorial logistic and Poisson regression analyses were used to identify determinants of overnutrition and undernutrition. The results showed a distinct profile compared with national averages: wasting was more prevalent (9·6 %), while overweight (8·6 %) and obesity (5·0 %) were less common. Notably, hyperuricaemia (27·6 %) and Zn deficiency (17·9 %) were elevated, whereas classical metabolic syndrome (2·3 %) was lower, delineating a regional pattern that prioritises these emerging and micronutrient issues. Marginal vitamin A deficiency (17·2 %) and vitamin D inadequacy (50·9 %) remained significant. Older age (11–17 years) was a strong risk factor for overnutrition-related disorders and hyperuricaemia (All *P* < 0·001). Overweight/obesity increased risks of hyperuricaemia and metabolic syndrome (All *P* < 0·001). Being female was a major risk factor for undernutrition (prevalence ratio = 1·27, 95 % CI: 1·19, 1·35, *P* < 0·001) and vitamin D deficiency (adjusted OR = 2·51, 95 % CI: 2·10, 3·00, *P* < 0·001), but a protective factor against hyperuricaemia (adjusted OR = 0·34, 95 % CI: 0·27, 0·41, *P* < 0·001). Frequent sugary drink consumption (≥ 3/week) elevated hyperuricaemia risk (adjusted OR = 1·33, 95 % CI: 1·05, 1·69, *P* = 0·020). This study underscores a complex double burden of malnutrition in western China, characterised by specific priority areas, and necessitates tailored, multi-component interventions such as limiting sugary drinks and focusing nutrition support on adolescent girls.

Nutrition plays an important role in the growth and development of children and adolescents. Malnutrition is one of the major public health problems affecting the health of children and adolescents worldwide. Chronic malnutrition inhibits growth and brain development, leading to lowered resistance and an increased risk of disease^(1,2)^.

Most child deaths are due to malnutrition, and malnutrition of all kinds is prevalent in children and adolescents internationally. Vitamin A is essential for children’s growth and development, visual function and immune function^(3)^. A 2009 report by the WHO shows that vitamin A deficiency affects more than 190 million preschool children worldwide, with higher rates in developing countries^(4)^. A study in China showed that the rate of vitamin A deficiency among children and adolescents increased from 1·5 % in 2016 to 3·5 % in 2021^(5)^. A large-scale cross-sectional study conducted in China in 2015 revealed that 23·3 % children suffered from low vitamin D status (deficiency and insufficiency)^(6)^. In the same trend as Vitamin A, Vitamin D deficiency rates are also trending high. About 1 billion people worldwide are Vitamin D deficient, with children and adolescents being the most at-risk group^(7)^. Zn is an essential micronutrient for the growth and development of children and adolescents. A 2012 study showed that the rate of Zn deficiency among children and adolescents in China was 6·8 %^(8)^. The prevalence of Zn deficiency (17·9 %) in this study was significantly higher than that of clinical vitamin A deficiency (0·5 %), a phenomenon worthy of further investigation. This disparity likely stems from the fundamental differences in the physiological roles and assessment methods of these two nutrients. Vitamin A possesses substantial storage capacity in the liver, maintaining normal serum retinol levels until reserves are severely depleted. This may explain why, despite a relatively high subclinical deficiency rate (17·2 %), the clinical deficiency rate remains low^(9)^. In contrast, Zn lacks functional storage reservoirs and more directly reflects recent dietary intake and bioavailability. The region’s high-phytic acid staple diet is known to significantly inhibit Zn absorption, making Zn deficiency a more sensitive indicator of inadequate dietary quality in this population^(10,11)^. This discrepancy underscores the importance of assessing multiple micronutrients to fully grasp the scope of nutritional challenges.

Overnutrition is also a serious nutritional health problem, and the results of five consecutive national surveys of students’ physical fitness and health, conducted from 1995–2004, indicate that the nutritional health problems of children and adolescents in China have shifted from malnutrition to overnutrition^(12)^. Nutrition in children and adolescents has become even more important in light of the global epidemic of obesity, metabolic syndrome (MetS) and other metabolic risk factors. More than 100 million children are obese worldwide^(13)^.

MetS is a complex group of metabolic disorder syndromes in which the body’s metabolism of proteins, fats, carbohydrates and other substances is disturbed. Diagnosis of MetS according to the National Cholesterol Education Program-Adult Treatment Panel III revised criteria for children and adolescents 6–17 Years of Age^(14)^. MetS was defined when participants had three or more of five conditions: abdominal obesity, hypertriglyceridaemia, hypoglyceridaemia, elevated blood pressure and elevated fasting blood glucose Studies have shown that children with MetS and other metabolic risk factors may be at significantly higher risk of developing type 2 diabetes and other CVD in adulthood^(15)^. In 2020, the global prevalence of MetS in children was 2·8 %^(16)^, and China’s analysis of MetS in children and adolescents in eight provinces and cities in 2012 showed that the prevalence of metabolic group syndrome in children and adolescents aged 7–17 years was 3·2 %^(17)^, which was higher than the global level. The prevalence of metabolic syndrome varies significantly across different populations, primarily due to close associations with gender, race/ethnicity and socio-economic factors^(18)^. A 1991–2015 meta-analysis showed that the prevalence of overweight and obesity in Chinese children and adolescents is generally on the rise^(19)^. At the same time, the prevalence of hyperuricaemia in children and adolescents has been increasing every year, from 16·7 % to 24·8 % between 2009 and 2019^(20)^. The prevalence of hyperuricaemia is also influenced by multiple factors including gender, age, ethnicity, geographic region and lifestyle^(21)^. Despite these valuable studies, significant knowledge gaps persist. Most prior research in Guizhou and Western China has focused on single nutritional issues – either micronutrient status or obesity-related metrics – in isolation. There is a notable lack of a comprehensive assessment that captures the full spectrum of the dual burden of malnutrition within the same cohort. Furthermore, the socio-demographic and behavioural factors driving this complex nutritional transition in the specific context of Guizhou are not well characterised.

Therefore, this study aims to fill these gaps by conducting an integrated analysis of the dual burden of malnutrition among children and adolescents aged 6–17 years in Guizhou. Building upon but differing from previous regional research, our study will simultaneously quantify the prevalence of both undernutrition (vitamin A deficiency (VAD), vitamin D deficiency (VDD) and Zn deficiency) and overnutrition (overweight/obesity, MetS and hyperuricaemia) using recent surveillance data and employ multivariable models to identify the specific demographic, familial and dietary factors associated with these conditions. This comprehensive approach will provide a contemporary evidence base for crafting targeted interventions to address the unique nutritional challenges facing children in this underdeveloped region of western China.

## Materials and methods

### Study participants

This study is a secondary analysis of cross-sectional data from the 2016–2017 China Nutrition and Health Monitoring Program for Children and Lactating Women. This project categorises all administrative counties/county-level cities/districts in China into four tiers based on population size and the National Bureau of Statistics’ urban–rural classification standards: large cities, small and medium-sized cities, ordinary rural counties and impoverished rural counties^(22)^. A total of 275 survey sites (county-level administrative areas) were selected in 2016–2017 as nationally and provincially representative sites. These included thirty-one large cities, 101 small and medium-sized cities, ninety-seven ordinary rural counties and forty-six impoverished rural counties. Two townships/subdistricts were randomly selected from each survey site, and two villages/neighbourhood committees were then randomly selected from each township/subdistrict. Our study selected data from four survey sites in urban and rural areas of Guizhou Province, western China, within this project to investigate the nutritional and health status of school-age participants. The study design and sampling methods are described in the article by Dongmei *et al.* introducing the China Nutrition and Health Surveys from 1982 to 2017^(23)^.

Guizhou Province has nine survey sites, specifically Dafang County, Bijie City; Nanming District, Guiyang City; Shuicheng District, Liupanshui City; Danzhai County, Qiandongnan Prefecture; Kaili City, Qiandongnan Prefecture; Fuquan City, Qiannan Prefecture; Longli County, Qiannan Prefecture; Dejiang County, Tongren City and Sinan County, Tongren City. Each survey site randomly selected one class for grades 1–6 of elementary school, one class for the first year of elementary school, one class for the second year of primary school, one class for grades 1–2 of junior high school and one class for grades 1–2 of high school. Ten classes were selected in total, with twenty-eight students in each class, totaling 280 students, with equal numbers of male and female students. Replacements were allowed only from the same village/neighbourhood and similar households as those of the original participants. The replacement rate had to be less than 35 %. Finally, 2423 participants were included after excluding those who were unable to provide key information (e.g. socio-demographic characteristics, laboratory test information or anthropometric measurements).

### Data collection and measurements

The survey utilised structured household and individual questionnaires provided by the CDC project team, designed to collect demographic information from students and parents, as well as data on physical activity and screen time^(24)^. Dietary intake information was obtained through a 3-d 24-h dietary recall questionnaire survey. The questionnaires were administered face-to-face by trained interviewers. The selection of food groups for analysis was guided by the Chinese Dietary Guidelines for School-Age Children (2022)^(25)^, to reflect key components of a healthy diet. We assessed the consumption of the following core food groups known for their provision of essential nutrients: dietary vegetables, fruits, milk, beans, meats, eggs and nuts. Additionally, we specifically evaluated the intake of sugary drinks as a distinct group due to its established role as a risk factor for poor nutritional outcomes, including overnutrition and metabolic disorders, in the child and adolescent population.

Screen time refers to the time using an electronic screen every day. A low level of screen time was defined as < 2 h and a high level was defined as ≥ 2 h. An exercise level of 0–3 d per week was defined as a low physical activity level; an exercise level for more than 4 d per week was defined as a high physical activity level^(26)^.

Anthropometric measurements, including height, weight, waist circumference and blood pressure, are centrally performed by trained local CDC staff using standard methods. All measuring instruments meet the national measurement certification requirements. Measurement methods were all in accordance with the standard requirements of the People’s Republic of China Industry Standard – Anthropometric Methods for Human Health Monitoring (WS/T424-2013). All equipment used in the measurements were selected according to the guidance of the national programme team. Height and weight were measured without shoes and coats. Height measurements were taken using a Nantong TZG meter from China with an accuracy of 0·1 cm. Weighing equipment was an electronic scale (TANITA HD-390), with results accurate to 0·1 kg. Waist circumference was measured using a soft tape, which was placed at the midpoint between the bottom of the thoracic cavity and the uppermost edge of the iliac bone after the participant exhaled in a fasted state. The blood pressure was measured with an electronic sphygmomanometer (Omron HBP 1300). Blood pressure was measured three times at 5-min intervals for each participant, and the mean values were recorded.

Blood biochemical tests included blood glucose, lipids, uric acid, serum vitamin A, vitamin D and Zn. Six millilitre of fasting venous blood was drawn from participants in the morning and tested for biochemical and nutritional indicators according to standard methods provided by the CDC project team.

### Diagnostic criteria and definitions

Nutritional status was screened according to the ‘Screening for Malnutrition in School-Aged Children and Adolescents’^(27)^. It is noteworthy that this Chinese standard utilises age- and sex-specific BMI cut-off points derived from national survey data, which differ from the Z-score criteria based on the WHO Growth Reference for the same age group. This distinction should be considered when comparing prevalence rates internationally^(28)^. In Table A.1 of the “Screening for Malnutrition in School aged Children and Adolescents”^(27)^, in addition to screening positive for growth retardation, screening for emaciation is also conducted based on Table B.1; The combination of growth retardation and emaciation is considered malnutrition. Refer to ‘Screening for Overweight and Obesity in School-Aged Children and Adolescents’^(29)^ for screening for overweight and obesity. Similarly, the screening thresholds for overweight and obesity in this standard (WS/T 586-2018) are based on Chinese national BMI percentiles, which are not directly equivalent to the WHO reference cut-offs (e.g. +1 sd and +2 sd for overweight and obesity, respectively), potentially leading to variations in prevalence estimates^(28)^.

Metabolic syndrome (MetS) was diagnosed according to the National Cholesterol Education Program-Adult Treatment Panel III revised criteria for children and adolescents aged 6–17 years. MetS was defined when participants had three or more of the following five conditions^(11)^:

1. Abdominal obesity: a waist circumference ≥ 90th percentile for age and gender specificity^(30)^;
2. Elevated TAG: TAG ≥ 1·24 mmol/l;
3. Low HDL: HDL ≤ 1·03 mmol/l;
4. Elevated blood pressure: systolic blood pressure or diastolic blood pressure ≥ 90th percentile for gender, age and height^(31)^;
5. Elevated fasting blood glucose: glucose ≥ 6·1 mmol/l.

The diagnostic criteria and definitions in this study were as follows:

Elevated total cholesterol (TC): TC ≥ 5·18 mmol/l^(32)^; elevated LDL: LDL ≥ 3·36 mmol/l^(21)^;

Hyperuricaemia: serum uric acid ≥ 357 µmol/l^(33)^ ;

Vitamin A deficiency (VAD), vitamin A marginal deficiency (VAMD) and vitamin A sufficiency as defined by the ‘Screening Method for Vitamin A Deficiency in Population’: serum retinol < 0·2 µg/ml for deficiency, 0·2–0·3µg/ml for marginal deficiency, > 0·3µg/ml for sufficiency, respectively^(34)^. Both VAD and VAMD are classified as vitamin A insufficiency;

According to the ‘Evaluation of Vitamin D Nutritional Status and Expert Consensus’, serum 25(OH)D levels < 12 ng/ml are considered Vitamin D deficiency (VDD), 12–20 ng/ml are vitamin D inadequacy (VDI) and ≥ 20 ng/ml are vitamin D sufficiency^(35)^. Both VDD and VDI are classified as vitamin D insufficiency;

Zn deficiency in children and adolescents was defined as serum Zn concentrations < 76·5 µg/dl^(22)^.

This study follows the classic framework of the ‘dual burden of malnutrition’, analysing diseases related to overnutrition alongside those related to undernutrition. The number of overnutrition-related disorders was a composite score that included a count of the five individual MetS components (abdominal obesity, high TG, low HDL, elevated blood pressure and elevated fasting blood glucose) if present, plus the presence of hyperuricaemia, high LDL or high TC. Hyperuricaemia was classified as overnutrition related due to its association with obesity and dietary excess. The number of undernutrition-related disorders included a count of prevalent vitamin A deficiency, vitamin A marginal deficiency, vitamin D deficiency, inadequacy of vitamin D and Zn deficiency. This classification approach is supported by prior research. For instance, metabolic syndrome is widely recognised as a cluster of nutritional excess^(36)^, hyperuricaemia is closely associated with multiple metabolic abnormalities^(37)^, and deficiencies in various micronutrients are often assessed collectively as indicators of malnutrition^(38)^.

### Statistical analysis

All statistical analyses were performed using SPSS (version 25.0). The normality of quantitative data was assessed using the Kolmogorov–Smirnov test. Based on the normality of variable distributions, results are presented as mean (standard deviation, sd) or median (interquartile range, IQR), while categorical data are reported as counts (percentages). Comparisons of categorical variables (e.g. gender and age groups) between groups were conducted using Pearson’s *χ*^2^ test, for which the assumption of expected cell counts (≥ 5) was verified. To identify factors associated with nutritional outcomes, we employed multivariable regression models tailored to the outcome type. Multivariable logistic regression was used for specific binary outcomes (e.g. metabolic syndrome and specific micronutrient deficiencies) to calculate adjusted OR. Potential interaction effects between age and gender were tested by including their product term in the respective models. Model performance was assessed by Hosmer–Lemeshow test. For the composite category of ‘overnutrition’ and ‘undernutrition’ with high prevalence rates, an exploratory multivariate Poisson regression model with robust variance was employed to obtain an overview of the generalised burden and to calculate prevalence ratio (PR), as the data did not exhibit significant overdispersion. Variable selection was based on clinical relevance, and all variables reported in the literature were included. All models adjusted for age and sex, and no severe multicollinearity was identified with variance inflation factors (VIF) < 5. Furthermore, potential interaction between age and gender was tested in the models. Sensitivity analyses conducted by excluding participants with any missing data showed no substantial changes compared with the original analysis. Missing data in the analytical variables were handled using listwise deletion. Statistical significance was set at *α* = 0·05 (two-sided, *P* < 0·05).

## Results

### Characteristics of research populations

A total of 2423 students were surveyed, there were 1211 (50 %) males and 1212 (50 %) females; 1011 (41·7 %) were age 6–10 years group, 715 (29·5 %) were age 11–13 years group and 697 (28·8 %) were age 14–17 years group. In Table 1, age group, physical activity, age of mother, education of mother, education of father and household size were not significantly different between male and female participants (All *P* > 0·05). There were significant difference in screen time and age of father between genders (*P* = 0·043, *P* = 0·033).

**Table 1.** Basic characteristics of the research population Table 1 long description.

Variables	Males		Females		All		*t* or $\chi^{2}$ values	*P* values
	*n* 1211		*n* 1212		*n* 2423			
	*n*	%	*n*	%	*n*	%		
Age								
Mean	11·95		11·98		11·97		–0·263	0·792
sd	3·29		3·30		3·29			
Age group							0·389	0·823
6–10	500	41·3	511	42·2	1011	41·7		
11–13	363	30·1	350	28·9	715	29·5		
14–17	346	28·6	351	29·0	697	28·8		
Physical activity							0·627	0·428
Low	606	50·0	626	51·7	1232	50·8		
High	605	50·0	586	48·3	1191	49·2		
Screen time							0·043	0·876
Low	943	77·9	948	78·2	1891	78·0		
High	268	22·1	264	21·8	532	22·0		
Age of mother							0·182	0·670
≤ 37 (median)	602	49·7	613	50·6	1215	50·1		
≥ 38	609	50·3	599	49·4	1208	49·9		
Age of father							0·033	0·856
≤ 40 (median)	643	53·1	648	53·5	1291	53·3		
≥ 41	568	46·9	564	46·5	1132	46·7		
Education of mother							2·967	0·227
Primary school and below	593	49·0	634	52·3	1227	50·6		
Middle school	566	46·7	534	44·1	1100	45·4		
University and above	52	4·3	44	3·6	96	4·0		
Education of father							3·522	0·172
Primary school and below	482	39·8	522	43·1	1004	41·4		
Middle school	654	54·0	629	51·9	1283	53·0		
University and above	75	6·2	61	5·0	136	5·6		
Household size							12·068	0·001
≤ 4 members	647	53·4	562	46·4	1209	49·9		
≥ 5 members	564	46·6	650	53·6	1214	50·1		
Location							0·444	1·000
Dafang County	134	11·1	135	11·1	269	11·1		
Nanming District	137	11·3	137	11·3	274	11·3		
Shuicheng District	123	10·1	127	10·5	250	10·3		
Danzhai County	139	11·5	131	10·8	270	11·1		
Kaili City	132	10·9	136	11·2	268	11·1		
Fuquan City	136	11·2	133	11·0	269	11·1		
Longli County	140	11·6	138	11·4	278	11·5		
Dejiang County	132	10·9	132	10·9	264	10·9		
Sinan County	139	11·5	142	11·7	281	11·6		

Significant gender differences were observed in most nutritional and biochemical indicators, with specific magnitudes and directions detailed in Table 2. In anthropometric and blood pressure measures, males had significantly lower median height (142·0 *v*. 144·0 cm, *P* < 0·001) and DBP (63·7 *v*. 64·3 mmHg, *P* = 0·069), but higher weight (35·5 *v*. 37·4 kg, *P* = 0·039), waist circumference (61·1 *v*. 60·5 cm, *P* = 0·012) and systolic blood pressure (110·3 *v*. 107·3 mmHg, *P* < 0·001). Regarding biochemical profiles, males exhibited lower median levels of TAG (0·77 *v*. 0·88 mmol/l, *P* < 0·001), TC (3·53 *v*. 3·66 mmol/l, *P* < 0·001) and LDL (1·93 *v*. 2·00 mmol/l, *P* = 0·001). Conversely, males showed higher median levels of fasting blood glucose (4·77 *v*. 4·65 mmol/l, *P* < 0·001), serum uric acid (326·0 *v*. 290·0 μmol/l, *P* < 0·001), vitamin D (19·5 *v*. 16·6 ng/ml, *P* < 0·001) and Zn (90·0 *v*. 88·0 μg/dl, *P* = 0·002). No statistically significant differences were found for HDL and vitamin A (*P* = 0·823, *P* = 0·370).

**Table 2.** Nutritional and biochemistry indicators for the research population Table 2 long description.

Variables	Males		Females		All		*Z* values	*P* values
	Median	IQR	Median	IQR	Median	IQR		
Anthropometric measurements								
Height (cm)	142·0	130·0–161·1	144·0	129·0–153·0	143·0	129·6–155·3	–3·981	< 0·001
Weight (kg)	35·5	26·8–49·5	37·4	25·7–47·0	36·3	26·3–47·9	–2·065	0·039
WC (cm)	61·1	54·6–68·1	60·5	53·1–67·5	60·8	54·0–67·6	–2·504	0·012
SBP (mmHg)	110·3	102·0–118·0	107·3	99·7115·3	108·7	101·0–116·7	–5·011	< 0·001
DBP (mmHg)	63·7	58·0–69·3	64·3	58·7–70·0	64·0	58·3–69·7	–1·816	0·069
Biochemical indexes								
FBG (mmol/l)	4·77	4·39–5.12	4·65	4·29–5·02	4·71	4·33–5·08	–4·626	< 0·001
TAG (mmol/l)	0·77	0·62–1·04	0·88	0·71–1·13	0·83	0·66–1·09	–7·589	< 0·001
TC (mmol/l)	3·53	3·17–3·99	3·66	3·29–4·03	3·60	3·23–4·01	–4·336	< 0·001
HDL (mmol/l)	1·38	1·19–1·62	1·40	1·19–1·61	1·39	1·19–1·61	–0·224	0·823
LDL (mmol/l)	1·93	1·63–2·28	2·00	1·68–2·34	1·97	1·66–2·31	–3·239	0·001
Serum uric acid (µmol/l)	326·0	273·0–387·0	290·0	251·0–337·0	307·0	260·0–363·0	–11·246	< 0·001
Vitamin A (µg/ml)	0·39	0·32–0·46	0·39	0·33–0·47	0·39	0·33–0·47	–0·896	0·370
Vitamin D (ng/ml)	19·5	15·7–23·7	16·6	13·3–20·4	18·1	14·3–22·4	–11·354	< 0·001
Zn (µg/dl)	90·0	81·0–98·0	88·0	80·0–97·0	89·0	80·0–97·0	–3·045	0·002

IQR, interquartile range; WC, waist circumference; SBP, systolic blood pressure; DBP, diastolic blood pressure; FBG, fasting blood glucose; TC, total cholesterol.

### Nutritional and health status of school-age children and adolescents

**Figure 1.**
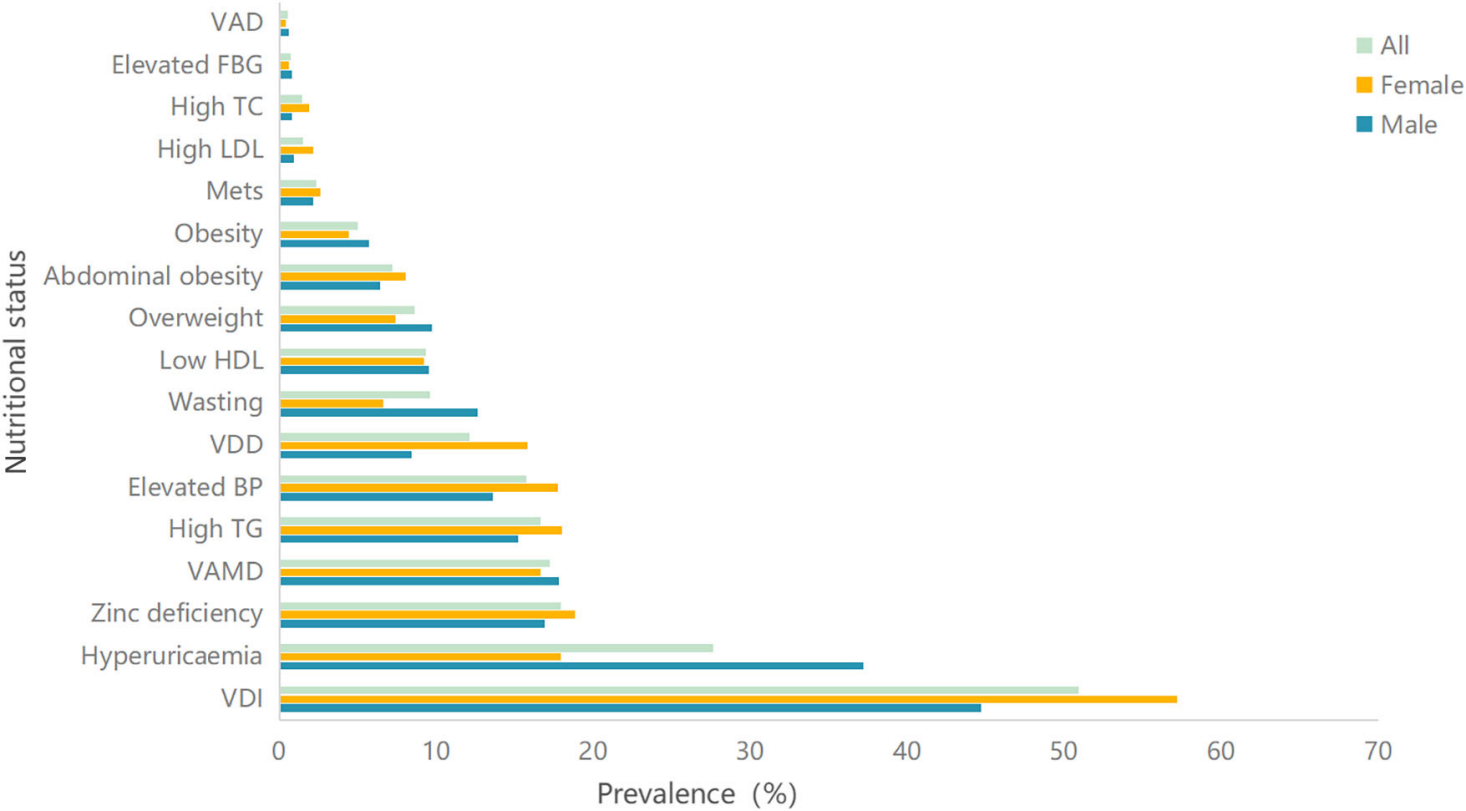
Sequence of prevalence of the relevant nutritional and health problems among school-age children and adolescents. VDI, vitamin D inadequacy; VAMD, vitamin A marginal deficiency; elevated BP, elevated blood pressure; VDD, vitamin D deficiency; MetS, metabolic syndrome; high TC, high total cholesterol; elevated FBG, elevated fasting blood glucose; VAD, vitamin A deficiency. MetS and its five components (central obesity, high TAG, low HDL, elevated BP and elevated FBG) were diagnosed according to the modified criteria of the National Cholesterol Education Program-Adult Treatment Panel III (NCEP-ATP III) for children and adolescents aged 6–17 years.

As illustrated in Figure 1, VDI was the most prevalent condition, affecting 50·9 % of the study population, followed by hyperuricaemia (27·6 %). Other notable conditions included Zn deficiency (17·9 %), marginal vitamin A deficiency (VAMD, 17·2 %), high TAG (16·6 %) and elevated blood pressure (15·7 %). The prevalence of other nutritional disorders, such as vitamin D deficiency (VDD, 12·1 %), wasting (9·6 %) and metabolic syndrome (MetS, 2·3 %), was also observed. The full spectrum of prevalence rates for all assessed conditions is detailed in Figure 1. In terms of gender, the top three prevalent nutrition-related diseases among male are VDI, hyperuricaemia and VAMD, with prevalence rates of 44·7 %, 37·2 % and 17·8 %, respectively; among female, the top three prevalent nutrition-related diseases are VDI, Zn deficiency and high TG, with prevalence rates of 57·2 %, 18·8 % and 18 %, respectively. There were significant differences between genders in the prevalence of weight groups, elevated blood pressure, high TC, high LDL, hyperuricaemia and vitamin D conditions, as shown in Table 3.

**Table 3.** Prevalence of various categories of nutritional status among school-age children and adolescents in Western China Table 3 long description.

Nutritional status	Males		Females		Total		$\chi^{2}$ Values	*P* values
	*n*	%	*n*	%	*n*	%		
Weight groups							33·600	< 0·001
Wasting	145	12·6	76	6·6	221	9·6		
Normal weight	829	72·0	933	81·6	1762	76·8		
Overweight	112	9·7	85	7·4	197	8·6		
Obesity	65	5·7	49	4·4	114	5·0		
MetS							0·653	0·419
No	1186	97·9	1181	97·4	2367	97·7		
Yes	25	2·1	31	2·6	56	2·3		
Abdominal obesity							2·207	0·137
No	1133	93·6	1115	92·0	2248	92·8		
Yes	78	6·4	97	8·0	175	7·2		
Elevated BP							7·754	0·005
No	1046	86·4	997	82·3	2043	84·3		
Yes	165	13·6	215	17·7	380	15·7		
Elevated FBG							0·536	0·464
No	1201	99·2	1205	99·4	2406	99·3		
Yes	10	0·8	7	0·6	17	0·7		
High TC							5·181	0·023
No	1201	99·2	1189	98·1	2390	98·5		
Yes	10	0·8	23	1·9	33	1·4		
High TG							3·414	0·065
No	1027	84·8	994	82·0	2021	83·4		
Yes	184	15·2	218	18·0	402	16·6		
Low HDL							0·082	0·775
No	1096	90·5	1101	90·8	2197	90·7		
Yes	115	9·5	111	9·2	226	9·3		
High LDL							5·515	0·019
No	1200	99·1	1187	97·9	2387	98·5		
Yes	11	0·9	25	2·1	36	1·5		
Hyperuricaemia							113·437	< 0·001
No	760	62·8	995	82·1	1755	72·4		
Yes	451	37·2	217	17·9	668	27·6		
Vitamin A							0·949	0·622
Sufficiency	989	81·7	1006	83·0	1995	82·3		
Marginal deficiency	215	17·8	201	16·6	416	17·2		
Deficiency	7	0·6	5	0·4	12	0·5		
Vitamin D							110·042	< 0·001
Sufficiency	568	46·9	328	27·1	896	37·0		
Inadequacy	541	44·7	693	57·2	1234	50·9		
Deficiency	102	8·4	191	15·8	293	12·1		
Zn deficiency							1·465	0·226
No	1006	83·1	984	81·2	1990	82·1		
Yes	205	16·9	228	18·8	433	17·9		

MetS, metabolic syndrome; elevated BP, elevated blood pressure; elevated FBG, elevated fasting blood glucose; high TC, high total cholesterol.

### Dietary intake of school-age children and adolescents

As shown in Table 4, among the eight food groups assessed, the intake of sugary drinks was the only one that showed a statistically significant difference between genders (*P* < 0·05). No significant differences were observed for the other food groups, including beans, milk, meats, eggs, vegetables, fruits and nuts (All *P* > 0·05).

**Table 4.** Dietary intake of the research population Table 4 long description.

Variables	Males		Females		All		$\chi^{2}$ Values	*P* values
	*n*	%	*n*	%	*n*	%		
Dietary beans							0·460	0·498
< 3/week	707	58·4	724	59·7	1431	59·1		
≥ 3/week	504	41·6	488	40·3	992	40·9		
Dietary milk							1·714	0·191
< 3/week	894	73·8	866	71·5	1760	72·6		
≥ 3/week	317	26·2	346	28·5	663	27·4		
Dietary meats							1·159	0·282
< 3/week	924	76·3	947	78·1	1871	77·2		
≥ 3/week	287	23·7	265	21·9	552	22·8		
Dietary eggs							0·003	0·954
< 3/week	883	72·9	885	73·0	1768	73·0		
≥ 3/week	328	23·1	327	27·0	655	27·0		
Sugary drink							11·047	0·001
< 3/week	917	75·7	985	81·3	1902	78·5		
≥ 3/week	294	24·3	227	18·7	521	21·5		
Dietary vegetables							0·480	0·489
< 3/week	1135	93·7	1144	94·4	2279	94·1		
≥ 3/week	76	6·3	68	5·6	144	5·9		
Dietary fruits							0·039	0·844
< 3/week	777	64·2	773	63·8	1550	64·0		
≥ 3/week	434	35·8	439	36·2	873	36·0		
Dietary nuts							0·005	0·946
< 3/week	1053	87·0	1055	87·0	2108	87·0		
≥ 3/week	158	13·0	157	13·0	315	13·0		

### Related factors associated with various nutritional diseases

As shown in Table 5, the results of multivariate logistic regression analysis showed that overweight and obesity were risk factors for MetS compared with the reference group (OR = 21·76, 95 % CI: 10·29, 46·03, *P* < 0·001 and OR = 50·32, 95 % CI: 23·08, 109·74, *P* < 0·001). Compared with age of mother ≤ 37 years old, age of mother ≥ 38 years old was a protective factor for MetS (OR = 0·46, 95 % CI: 0·21, 0·99, *P* = 0·047), and education of mother in middle school was a risk factor for MetS (OR = 2·43, 95 % CI: 1·17, 5·017, *P* = 0·017), compared with education of mother in primary school and below, and compared with education of father in primary school and below, education of father in middle school was a protective factor for MetS (OR = 0·25, 95 % CI: 0·12, 0·52, *P* < 0·001).

When focusing on hyperuricaemia, a participant’s sex, age group, weight group, age of mother and sugary drink were the relevant factors. Female were less likely to have hyperuricaemia than males (OR = 0·34, 95 % CI: 0·27, 0·41, *P* < 0·001). Participants in 11–13 years and 14–17 years were more likely to have hyperuricaemia than 6–10 years (OR = 2·85, 95 % CI: 2·19, 3·70, *P* < 0·001, and OR = 4·74, 95 % CI: 3·55, 6·33, *P* < 0·001), overweight and obesity were more likely to have hyperuricaemia compared with the reference group (OR = 3·69, 95 % CI: 2·40, 5·66, *P* < 0·001 and OR = 1·75, 95 % CI: 1·25, 2·45, *P* = 0·001), mother’s age ≥ 38 years was more likely to have hyperuricaemia than those ≤ 37 years old (OR = 1·65, 95 % CI: 1·27, 2·15, *P* < 0·001) and frequency of sugary drink intake ≥ 3/week was more likely to have hyperuricaemia than < 3/week (OR = 1·33, 95 % CI: 1·05, 1·69, *P* = 0·020).

We found that participants aged 11–13 and 14–17 years (OR = 0·36, 95 % CI: 0·28, 0·48, *P* < 0·001, and OR = 0·13, 95 % CI: 0·09, 0·20, *P* < 0·001), obesity (OR = 0·40, 95 % CI: 0·20, 0·80, *P* = 0·009), and with fathers educated in middle school and university and above (OR = 0·66, 95 % CI: 0·51, 0·87, *P* = 0·003, and OR = 0·26, 95 % CI: 0·10, 0·70, *P* = 0·007) were less likely to have vitamin A insufficiency compared with the corresponding reference groups. Furthermore, significant age-by–sex interactions were identified. The protective effect of being female was significantly stronger in the youngest group, as evidenced by the interaction terms: for ages 6–10 × female, OR = 0·55, *P* = 0·019; and for ages 11–13 × female, OR = 0·15, *P* < 0·001 (reference: males aged 14–17).

With respect to vitamin D deficiency, a participant’s sex, age group, weight group and dietary nuts were the relevant factors. Compared with the corresponding reference group, females (OR = 2·51, 95 % CI: 2·10, 3·00, *P* < 0·001), 11–13 years, 14–17 years (OR = 2·20, 95 % CI: 1·77, 2·73, *P* < 0·001, and OR = 2·20, 95 % CI: 1·71, 2·83, *P* < 0·001), nut intake ≥ 3/week (OR = 1·41, 95 % CI: 1·08, 1·85, *P* = 0·013) was a risk factor for vitamin D deficiency. A significant interaction was also found between age and sex. Specifically, the interaction term for ages 11–13 × female was significant (OR = 1·69, *P* = 0·017), indicating that the risk of vitamin D deficiency associated with female sex was particularly elevated in this age group (reference: males aged 14–17 years).

Participants aged 11–13 years were less likely to be Zn deficiency compared with the reference group (OR = 0·73, 95 % CI: 0·55, 0·96, *P* = 0·010), and participants with higher levels of screen time were more likely to be Zn deficiency compared with those with lower levels of screen time (OR = 1·66, 95 % CI: 1·30, 2·12, *P* < 0·001). Those participants whose household size was ≥ 5 members were more prone to have Zn deficiency than were those whose household size was ≤ 4 members (OR = 1·34, 95 % CI: 1·01, 1·68, *P* = 0·010). Participants with ≥ 3/week were less likely to have Zn deficiency compared with those with a milk intake < 3/week (OR = 0·53, 95 % CI: 0·40, 0·70, *P* < 0·001). Participants with ≥ 3/week were less likely to have Zn deficiency compared with those with a sugary drink intake < 3/week (OR = 0·69, 95 % CI: 0·51, 0·92, *P* = 0·013). Participants with a frequency of vegetable and nut intake ≥ 3/week had a higher risk of Zn deficiency compared with the reference group, the OR were 1·98 and 1·55, respectively.

Model fit was assessed using the Hosmer–Lemeshow test, yielding the following results: MetS ( χ ^2^ = 1·715, *P* = 0·989), hyperuricaemia ( χ ^2^ = 35·844, *P* < 0·05), vitamin A insufficiency ( χ ^2^ = 6·999, *P* = 0·537), vitamin D insufficiency ( χ ^2^ = 6·719, *P* = 0·567) and Zn deficiency ( χ ^2^ = 3·960, *P* = 0·861). Except for the hyperuricaemia model, all models demonstrated good fit. (*P* > 0·05).

### The relevant factors of overnutrition- and undernutrition-related disorders

To provide a synthesised overview of the dual burden, we also conducted exploratory analyses using composite outcomes. We included metabolic syndrome and its five components (abdominal obesity, high TG, low HDL, elevated blood pressure and fasting blood glucose), hyperuricaemia, high LDL and high TC in the overnutrition-related disorders, while vitamin A insufficiency, vitamin D deficiency and Zn deficiency were included in the undernutrition-related disorders. Multivariate Poisson regression analysis was used to test the associations of demographic information, parental characteristics and family characteristics, physical activity and screen time with them.

As shown in Table 6, age, weight, physical activity and household size were associated with overnutrition-related disorders, with the older age group (11–17 years), overweight and obesity being positively associated with overnutrition-related disorders (all PR > 1, and all *P* < 0·05), in contrast, participants with higher physical activity and household size ≥ 5 members were negatively associated with overnutrition-related disorders (All PR < 1, and all *P* < 0·05). In terms of undernutrition-related disorders, participants who were female and higher screen time were more likely to have undernutrition-related disorders (All PR > 1, and all *P* < 0·05), and participants whose fathers had higher levels of education had a lower risk of undernutrition-related disorders (all PR < 1, and all *P* < 0·05).

**Table 5.** Multivariate logistic regression analysis of various nutritional disorders among school-age children and adolescents in Western China Table 5 long description.

Characteristics	Metabolic syndrome			Hyperuricaemia			Vitamin A insufficiency			Vitamin D insufficiency			Zinc deficiency		
	AOR	95 % CI	*P* values	AOR	95 % CI	*P* values	AOR	95 % CI	*P* values	AOR	95 % CI	*P* values	AOR	95 % CI	*P* values
Sex															
Males (reference)	1·00		–	1·00		–	1·00		–	1·00		–	1·00		–
Females	1·63	0·89, 2·99	0·110	0·34	0·27, 0·41	< 0·001	0·86	0·69, 1·07	0·182	2·51	2·10, 3·00	< 0·001	1·11	0·89, 1·37	0·352
Age group															
6–10 (reference)	1·00		–	1·00		–	1·00		–	1·00		–	1·00		–
11–13	1·34	0·63, 2·84	0·448	2·85	2·19, 3·70	< 0·001	0·36	0·28, 0·48	< 0·001	2·20	1·77, 2·73	< 0·001	0·73	0·55, 0·96	0·024
14–17	1·48	0·63, 3·48	0·367	4·74	3·55, 6·33	< 0·001	0·13	0·09, 0·20	< 0·001	2·20	1·71, 2·83	< 0·001	1·04	0·77, 1·41	0·782
Weight group															
Others	1·00		–	1·00		–	1·00		–	1·00		–	1·00		–
Overweight	21·76	10·29, 46·03	< 0·001	3·69	2·40, 5·66	< 0·001	0·63	0·40, 1·00	0·050	1·31	0·95, 1·82	0·104	1·20	0·83, 1·75	0·339
Obesity	50·32	23·08, 109·74	< 0·001	1·75	1·25, 2·45	0·001	0·40	0·20, 0·80	0·009	1·32	0·86, 2·00	0·202	0·68	0·37, 1·24	0·205
Physical activity															
Low	1·00		–	1·00		–	1·00		–	1·00		–	1·00		–
High	1·41	0·72, 2·78	0·280	0·86	0·70, 1·05	0·134	1·21	0·97, 1·51	0·097	1·02	0·85, 1·21	0·858	0·90	0·72, 1·12	0·333
Screen time															
Low	1·00		–	1·00		–	1·00		–	1·00		–	1·00		–
High	1·40	0·76, 2·60	0·371	0·97	0·76, 1·23	0·786	1·00	0·76, 1·33	0·987	1·17	0·94, 1·45	0·153	1·66	1·30, 2·12	< 0·001
Age of mother															
≤ 37	1·00		–	1·00		–	1·00		–	1·00		–	1·00		–
≥ 38	0·46	0·21, 0·99	0·047	1·65	1·27, 2·15	< 0·001	1·11	0·83, 1·49	0·486	0·84	0·67, 1·06	0·140	1·03	0·77, 1·40	0·825
Age of father															
≤ 40	1·00		–	1·00		–	1·00		–	1·00		–	1·00		–
≥ 41	1·90	0·87, 4·16	0·109	1·01	0·78, 1·31	0·931	0·77	0·58, 1·05	0·096	1·15	0·92, 1·45	0·220	0·77	0·58, 1·03	0·074
Education of mother															
Primary school and below	1·00		–	1·00		–	1·00		–	1·00		–	1·00		–
Middle school	2·43	1·17, 5·01	0·017	1·10	0·86, 1·40	0·442	0·84	0·64, 1·12	0·232	1·04	0·84, 1·30	0·702	0·91	0·70, 1·19	0·499
University and above	–		–	0·98	0·50, 1·93	0·953	0·52	0·17, 1·64	0·266	0·89	0·48, 1·64	0·704	0·53	0·17, 1·64	0·267
Education of father															
Primary school and below	1·00		–	1·00		–	1·00		–	1·00		–	1·00		–
Middle school	0·25	0·12, 0·52	< 0·001	1·00	0·79, 1·26	0·980	0·66	0·51, 0·87	0·003	0·91	0·73, 1·12	0·356	0·87	0·67, 1·12	0·282
University and above	0·21	0·04, 1·20	0·079	1·33	0·74, 2·40	0·345	0·26	0·10, 0·70	0·007	1·14	0·66, 1·95	0·646	0·44	0·18, 1·11	0·083
Household size															
≤ 4 members	1·00		–	1·00		–	1·00		–	1·00		–	1·00		–
≥ 5 members	0·66	0·35, 1·23	0·187	0·90	0·74, 1·11	0·338	1·15	0·91, 1·44	0·241	1·00	0·83, 1·19	0·949	1·34	1·01, 1·68	0·010
Dietary beans															
< 3/week	1·00		–	1·00		–	1·00		–	1·00		–	1·00		–
≥ 3/week	0·83	0·44, 1·55	0·557	0·85	0·69, 1·04	0·119	1·20	0·96, 1·51	0·112	1·05	0·88, 1·26	0·576	1·17	0·94, 1·47	0·161
Dietary milk															
< 3/week	1·00		–	1·00		–	1·00		–	1·00		–	1·00		–
≥ 3/week	1·43	0·72, 2·81	0·306	1·14	0·90, 1·44	0·269	1·05	0·80, 1·37	0·744	1·08	0·87, 1·32	0·496	0·53	0·40, 0·70	< 0·001
Dietary meats															
< 3/week	1·00		–	1·00		–	1·00		–	1·00		–	1·00		–
≥ 3/week	1·34	0·64, 2·79	0·442	1·10	0·86, 1·40	0·454	0·75	0·54, 1·03	0·072	1·15	0·92, 1·44	0·232	0·92	0·69, 1·22	0·560
Dietary eggs															
< 3/week	1·00		–	1·00		–	1·00		–	1·00		–	1·00		–
≥ 3/week	0·84	0·42, 1·68	0·617	0·97	0·77, 1·22	0·805	0·95	0·74, 1·23	0·705	1·02	0·84, 1·25	0·835	1·13	0·88, 1·45	0·332
Sugary drink															
< 3/week	1·00		–	1·00		–	1·00		–	1·00		–	1·00		–
≥ 3/week	0·66	0·29, 1·54	0·339	1·33	1·05, 1·69	0·020	1·08	0·80, 1·46	0·602	0·90	0·72, 1·12	0·338	0·69	0·51, 0·92	0·013
Dietary vegetables															
< 3/week	1·00		–	1·00		–	1·00		–	1·00		–	1·00		–
≥ 3/week	0·49	0·10, 2·55	0·400	1·15	0·76, 1·72	0·515	1·53	0·98, 2·37	0·060	1·05	0·72, 1·52	0·817	1·98	1·33, 2·96	0·001
Dietary fruits															
< 3/week	1·00		–	1·00		–	1·00		–	1·00		–	1·00		–
≥ 3/week	0·86	0·44, 1·68	0·656	1·10	0·89, 1·36	0·381	0·97	0·77, 1·22	0·782	0·92	0·76, 1·10	0·355	0·85	0·67, 1·07	0·169
Dietary nuts															
< 3/week	1·00		–	1·00		–	1·00		–	1·00		–	1·00		–
≥ 3/week	1·41	0·64, 3·11	0·390	1·03	0·76, 1·39	0·853	1·07	0·77, 1·48	0·694	1·41	1·08, 1·85	0·013	1·55	1·15, 2·10	0·004

AOR,adjusted OR.

**Table 6.** Multivariate Poisson regression analysis of overnutrition- and undernutrition-related disorders among school-age children and adolescents in Western China Table 6 long description.

	Overnutrition-related disorders			Undernutrition-related disorders		
	PR	95 % CI	*P* values	PR	95 % CI	*P* values
Characteristics						
Sex						
Males (reference)	1·00		–	1·00		–
Females	0·94	0·86, 1·02	0·144	1·27	1·19, 1·35	< 0·001
Age group						
6–10 (reference)	1·00		–	1·00		–
11–13	1·51	1·35, 1·69	< 0·001	1·06	0·98, 1·14	0·167
14–17	1·77	1·56, 2·00	< 0·001	1·02	0·93, 1·11	0·684
Weight group						
Others	1·00		–	1·00		–
Overweight	2·04	1·81, 2·31	< 0·001	1·00	0·89, 1·12	0·989
Obesity	3·47	3·09, 3·90	< 0·001	0·95	0·81, 1·10	0·461
Physical activity						
Low	1·00		–	1·00		–
High	0·89	0·82, 0·97	0·010	1·03	0·97, 1·10	0·391
Screen time						
Low	1·00		–	1·00		–
High	1·03	0·92, 1·14	0·641	1·13	1·05, 1·21	0·002
Age of mother						
≤ 37	1·00		–	1·00		–
≥ 38	1·07	0·95, 1·14	0·281	0·99	0·91, 1·07	0·718
Age of father						
≤ 40	1·00		–	1·00		–
≥ 41	1·06	0·94, 1·21	0·316	0·97	0·90, 1·06	0·510
Education of mother						
Primary school and below	1·00		–	1·00		–
Middle school	1·08	0·97, 1·19	0·165	0·98	0·91, 1·06	0·626
University and above	0·93	0·71, 1·23	0·621	0·98	0·78, 1·22	0·838
Education of father						
Primary school and below	1·00		–	1·00		–
Middle school	0·92	0·83, 1·02	0·097	0·90	0·83, 0·97	0·005
University and above	0·91	0·69, 1·20	0·515	0·80	0·66, 0·96	0·016
Household size						
≤ 4 members	1·00		–	1·00		–
≥ 5 members	0·89	0·81, 0·97	0·008	1·06	1·00, 1·13	0·072

PR, prevalence ratios.

Overnutrition-related disorders: the prevalent five MetS components and hyperuricaemia, high LDL, and high TC. Undernutrition-related disorders: prevalent vitamin A insufficiency, vitamin D deficiency, and Zn deficiency.

For the Poisson regression models, the ratio of the scaled Pearson *χ*^2^ statistic to its degrees of freedom was close to 1 (0·936 for overnutrition-related disorders and 0·694 for undernutrition-related disorders), indicating no significant overdispersion and thus supporting the appropriateness of the Poisson regression approach.

## Discussion

This study reveals a complex and regionally distinct profile of the double burden of malnutrition among children and adolescents in Guizhou Province, western China. Moving beyond the simple coexistence of undernutrition and overnutrition, our analysis identifies three critical and interrelated public health priorities that demand urgent, targeted intervention: a notably high prevalence of hyperuricaemia (27·6 %) with clear behavioural determinants, a severe and gender-skewed burden of vitamin D inadequacy (50·9 %) concentrated in adolescent girls and a regional Zn deficiency crisis (17·9 %) linked to local dietary patterns.

Hyperuricaemia emerged as the most prevalent metabolic disorder in our study, exceeding the previously reported national average of 23·3 % for Chinese children and adolescents^(20)^. Multivariate analysis confirmed that being male, older age (11–17 years), overweight/obesity, older maternal age (≥ 38 years) and frequent consumption of sugar-sweetened beverages (≥ 3 times per week) were all significant risk factors. This is in keeping with previous research that males, who are overweight and obesity, are more likely to develop hyperuricaemia^(39,40)^. Many studies have shown that obesity is a risk factor for hyperuricaemia, and its pathogenic mechanisms include lipid dysregulation, insulin resistance, inflammation and adipokine imbalance^(41,42)^. Therefore, maintaining healthy weight is an effective measure for children and adolescents to prevent hyperuricaemia. The mechanism of the sex difference in hyperuricaemia is not well understood, but the difference may be explained by sex hormones, such as the role of oestrogen on xanthine oxidase activity and renal excretion of uric acid^(43)^. Notably, our analysis revealed a significant interaction between age and sex, indicating that the protective effect of female sex was most pronounced in early childhood and attenuated with advancing age. This dynamic pattern further strengthens the hypothesis that the onset of puberty and its associated hormonal changes are key modifiers of hyperuricaemia risk. Muscle tissue is the main site of de novo purine production in the body, and muscle mass increases more during puberty. Thus, the difference in muscle mass between males and females may be another explanation for the difference in prevalence between males and females during puberty^(44)^. Notably, the prevalence of hyperuricaemia in our study (27·6 %) is higher than the previously reported national average of 23·3 % for Chinese children and adolescents but considerably lower than the 50·4 % reported in Sichuan province^(20)^. This regional variation may be partly attributable to differences in dietary patterns, particularly the intake of sugar-sweetened beverages^(45)^. Sugar-sweetened beverages are usually high in fructose, and studies have shown that fructose-containing sweeteners and beverages increase the risk of hyperuricaemia by stimulating the catabolism of adenine nucleotides, the metabolism of fructose uniquely promotes uric acid generation by accelerating adenosine triphosphate depletion and adenine nucleotide catabolism^(46)^. Therefore, controlling the intake of sugary drink is beneficial in reducing the prevalence of hyperuricaemia in children and adolescents. This pattern underscores that preventive strategies, such as public health campaigns to reduce sugar-sweetened beverages consumption and school-based weight management programmes, should be initiated before and intensively during adolescence, with particular emphasis on boys.

The alarmingly high rate of VDI represents a profound public health concern, with adolescent girls bearing a disproportionate burden. Vitamin D inadequacy and deficiency (< 20 ng/ml for VDI, < 12 ng/ml for VDD) were 50·9 % and 12·1 % among school-aged children and adolescents in this study, respectively, and were higher in females (VDI: 57·2 %, VDD: 15·8 %) than males (44·7 %, 8·4 %). The significant age-by-sex interaction, showing peak risk among 11–13-year-old girls (interaction OR = 1·69), pinpoints early adolescence as a critical window where gender disparity amplifies. This finding can be attributed to a perfect storm of behavioural and social factors converging at this life stage. Biologically, the growth spurt increases demand. Behaviourally, girls at this age often develop a stronger preference for indoor or shaded activities, and both parents and the girls themselves exhibit heightened concern about skin whitening and sun protection, leading to more consistent use of sunscreen^(47)^. Physiologically, sunscreen effectively blocks UVB radiation, which is essential for cutaneous vitamin D synthesis. The key physiological explanation lies in the fact that sunscreen effectively blocks UVB radiation (290–315 nm), which is exclusively responsible for the photoconversion of 7-dehydrocholesterol to pre-vitamin D₃ in the skin^(48)^. While experimental studies under ideal conditions show sunscreen can block over 90 % of vitamin D synthesis^(49)^, its real-world impact is likely modulated by typical usage patterns where people apply insufficient amounts^(50)^. Socially, increasing academic demands drastically reduces opportunities for outdoor activity during daylight hours^(51)^. The link with nut intake, while unexpected, might be related to dietary composition or confounding by lifestyle and warrants further investigation. To address the high prevalence of vitamin D insufficiency, our findings suggest a shift in public health strategy is needed. For adolescents, especially early adolescent girls, promoting sensible sun exposure (e.g. 10–30 min of midday sun exposure on arms and legs several times a week without sunscreen) is crucial^(52)^. Concurrently, revising and enforcing vitamin D supplementation guidelines for school-age children and adolescents, alongside mandatory food fortification programmes, are essential evidence-based measures to bridge this nutrient gap.

The prevalence of Zn deficiency in our study (17·9 %) was substantially higher than the reported national average of 6·8 %, signalling a specific regional micronutrient gap^(8)^. The associated risk factors reveal a telling tension with local diets. Frequent intake of vegetables and nuts – staples of a plant-based diet – was associated with increased risk. This is likely attributable to their high phytic acid (myo-inositol hexakisphosphate) content, a potent chelator that binds Zn in the gut to form insoluble complexes, significantly inhibiting its absorption^(53)^. Conversely, frequent milk consumption, a source of highly bioavailable Zn, was protective^(54)^. This suggests that traditional, plant-heavy dietary patterns, prevalent in regions like Guizhou where diets are reliant on rice and wheat (both high in phytates)^(55)^, in the absence of sufficient bioavailable Zn from animal-source foods, may inadvertently contribute to deficiency. The unexpected inverse association with sugary drink intake warrants caution and likely reflects residual confounding. Furthermore, the significant risk posed by higher screen time may be a proxy for a broader sedentary lifestyle, potentially associated with poorer overall dietary quality and irregular meal patterns, which could indirectly limit Zn intake. To address this high burden of deficiency, public health strategies should extend beyond general dietary advice. Specifically, promoting the consumption of Zn-rich dairy products, employing food fortification programmes in staple foods and advocating for dietary diversification with Zn-enhanced or low-phytate crop varieties represent targeted, evidence-based interventions to improve Zn status in this vulnerable population.

Our findings collectively argue for a decisive shift from generic nutrition guidelines to a stratified, evidence-based intervention framework. The dual burden in this region manifests not as equal weights but as a targetable syndemic with age- and sex-specific profiles. Crucially, the coexistence of markedly divergent micronutrient profiles – specifically, a very low prevalence of clinical vitamin A deficiency (0·5 %) alongside a high prevalence of Zn deficiency (17·9 %) – paints a nuanced picture of dietary patterns. This contrast suggests that local diets, potentially richer in provitamin A carotenoids from vegetables and pork (common in the regional ‘Yangtze River Basin’ dietary pattern)^(56)^, may be protective against severe VAD, while still being insufficient in bioavailable Zn due to high phytate content. Furthermore, the relatively low to moderate prevalence of classical cardiometabolic risk factors – such as hypertension (15·7 %), high LDL-cholesterol (1·5 %) and metabolic syndrome (2·3 %) – alongside the lower rates of overweight/obesity compared with national figures^(57)^, contrasts sharply with the alarmingly high rates of hyperuricaemia and general vitamin D inadequacy (50·9 %). This distinctive pattern confirms that Guizhou is in a unique, early phase of nutritional and epidemiological transition. The population is grappling with a complex mix: persistent undernutrition of specific micronutrients (Zn), emerging diet-driven overnutrition problems (hyperuricaemia), while being partly protected from classical deficiencies (clinical VAD) and not yet experiencing the full surge of obesity-related metabolic diseases. This early-phase profile offers a critical window for preventive action. In this context, the protective role of higher paternal education against undernutrition-related disorders and the benefits of higher physical activity and larger household size (≥ 5 members) against overnutrition-related disorders underscore the importance of socio-familial and behavioural levers. Therefore, effective strategies must be forward-looking yet context-specific, synergistically combining: Policy and regulatory actions (e.g. restricting sugar-sweetened beverages availability to curb hyperuricaemia and considering Zn/vitamin D fortification of staples); school-based precision programmes (e.g. targeted vitamin D supplementation for adolescent girls, nutrition education to promote Zn bioavailability) and family-engaged initiatives to empower caregivers, leveraging paternal education to improve household-level dietary quality.

This study has several limitations that should be acknowledged. First, the cross-sectional nature of the design precludes the establishment of causal relationships between the identified factors and the nutritional outcomes. Second, data on dietary and behavioural factors were collected via self-reported questionnaires, which are susceptible to recall bias and social desirability bias; furthermore, the non-quantitative nature of the dietary assessment limited our ability to precisely evaluate nutrient intake levels. Additionally, biochemical indicators such as vitamin D levels can be influenced by seasonal variation, a potential confounding factor that was not accounted for in this analysis. Furthermore, the external generalisability of our findings may be limited. Although the data were drawn from a surveillance programme with a complex, multi-stage sampling design, our analysis focused on a sub-sample from four specific counties in Guizhou Province and did not incorporate sampling weights. Therefore, the results are most representative of this specific region in western China and should be extrapolated to other populations with different socioeconomic or ethnic compositions with caution. This study assessed vitamin A deficiency based on serum retinol concentrations without adjusting for inflammatory markers and failed to measure more precise indicators such as retinol-binding protein or hepatic vitamin A stores. This may have impacted the accuracy of the estimated prevalence of vitamin A deficiency. Despite these limitations, the use of robust methodologies, including standardised physical examinations and laboratory tests, strengthens the internal validity of our findings regarding the complex nutritional landscape.

### Conclusions

This study delineates a distinct, early-phase double burden of malnutrition among children and adolescents in Guizhou Province, western China. Its core profile – prioritising hyperuricaemia, adolescent-girl-centric vitamin D inadequacy and diet-linked Zn deficiency over classical cardiometabolic risks – provides a clear map for public health action. These findings underscore the imperative to replace one-size-fits-all approaches with precision strategies, tailored to local dietary patterns and the specific age-sex vulnerabilities identified. Future efforts must integrate longitudinal research to establish causality and evaluate interventions, while policymakers should focus on creating synergistic actions across regulatory, community and household levels to effectively interrupt the intergenerational cycle of malnutrition in transitioning regions.

## Table 1 Long description

The table presents the basic characteristics of a research population of 2423 students, divided into males and females. It includes data on age, physical activity, screen time, age of mother, education of mother, education of father, household size, and location. The table has 12 columns and 20 rows, including headers and row labels. Column headers are Variables, Males n, Males %, Females n, Females %, All n, All %, t or χ² values, and P values. Row labels include Age, Age group, Physical activity, Screen time, Age of mother, Age of father, Education of mother, Education of father, Household size, and Location. Each row provides specific data points for males, females, and the total population, along with statistical values for comparison. Notable trends include the distribution of age groups, physical activity levels, screen time, and educational backgrounds of parents. The table indicates that most variables were not significantly different between male and female participants, except for screen time and age of father, which showed significant differences.

Navigate back to Table 1.

## Table 2 Long description

A table comparing nutritional and biochemical indicators between males and females. The table has 15 rows and 9 columns. The columns are labeled as Variables, Males Median, Males IQR, Females Median, Females IQR, All Median, All IQR, Z values, and P values. The rows are labeled as Height (cm), Weight (kg), WC (cm), SBP (mmHg), DBP (mmHg), FBG (mmol/l), TAG (mmol/l), TC (mmol/l), HDL (mmol/l), LDL (mmol/l), Serum uric acid (μmol/l), Vitamin A (μg/ml), Vitamin D (ng/ml), and Zn (μg/dl). Each row provides the median and interquartile range (IQR) values for males and females, as well as the overall median and IQR values. The table also includes Z values and P values for each variable.

Navigate back to Table 2.

## Table 3 Long description

The table presents the prevalence of various nutritional statuses among school-age children and adolescents in Western China, broken down by gender and overall totals. It includes data on weight groups, metabolic conditions, and vitamin deficiencies. The table has 15 rows and 10 columns, with column headers including Nutritional status, Males (n, %), Females (n, %), Total (n, %), and chi-square values with P values. Row labels include categories such as Weight groups, MetS, Abdominal obesity, Elevated BP, Elevated FBG, High TC, High TG, Low HDL, High LDL, Hyperuricaemia, Vitamin A, Vitamin D, and Zn deficiency. Each row provides the count and percentage of males, females, and the total population affected by each condition, along with chi-square values and P values indicating statistical significance. Notable trends include high prevalence rates of VDI and hyperuricaemia, with significant differences between genders in several conditions.

Navigate back to Table 3.

## Table 4 Long description

The table compares dietary intake across genders for various food groups. It has 8 rows and 8 columns. The columns are labeled as Variables, Males n, Males %, Females n, Females %, All n, All %, Chi-Squared Values, and P values. The rows are labeled as Dietary beans, Dietary milk, Dietary meats, Dietary eggs, Sugary drink, Dietary vegetables, Dietary fruits, and Dietary nuts. Each row provides the number and percentage of males and females consuming less than or greater than or equal to 3 servings per week, along with the total number and percentage for all participants. The Chi-Squared Values and P values are also provided for each food group. Notable trends include a significant difference in the intake of sugary drinks between genders, with a P value of 0.001, indicating statistical significance. No significant differences were observed for the other food groups, as indicated by P values greater than 0.05.

Navigate back to Table 4.

## Table 5 Long description

The table presents the results of a multivariate logistic regression analysis of various nutritional disorders among school-age children and adolescents in Western China. It includes characteristics such as sex, age group, weight group, physical activity, screen time, age of mother, age of father, education of mother, education of father, household size, and dietary habits. The table has 10 columns: Characteristics, Metabolic syndrome (AOR, 95% CI, P values), Hyperuricaemia (AOR, 95% CI, P values), Vitamin A insufficiency (AOR, 95% CI, P values), Vitamin D insufficiency (AOR, 95% CI, P values), and Zinc deficiency (AOR, 95% CI, P values). Row 1: Sex, Males (reference), Females (AOR 163, 95% CI 089, 299, P 0110). Row 2: Age group, 6-10 (reference), 11-13 (AOR 134, 95% CI 063, 284, P 0448), 14-17 (AOR 148, 95% CI 063, 348, P 0367). Row 3: Weight group, Normal (reference), Overweight (AOR 2176, 95% CI 1029, 4603, P < 0001), Obesity (AOR 5032, 95% CI 2308, 10974, P < 0001). Row 4: Physical activity, Low (reference), High (AOR 141, 95% CI 072, 278, P 0860). Row 5: Screen time, Low (reference), High (AOR 140, 95% CI 076, 260, P 0371). Row 6: Age of mother, < 37 (reference), >= 38 (AOR 046, 95% CI 021, 099, P 0047). Row 7: Age of father, < 40 (reference), >= 41 (AOR 190, 95% CI 087, 416, P 0109). Row 8: Education of mother, Primary school and below (reference), Middle school (AOR 243, 95% CI 117, 5017, P 0017), University and above (AOR 098, 95% CI 050, 193, P 0953). Row 9: Education of father, Primary school and below (reference), Middle school (AOR 025, 95% CI 012, 052, P < 0001), University and above (AOR 021, 95% CI 04, 120, P 079). Row 10: Household size, <= 4 members (reference), >= 5 members (AOR 066, 95% CI 035, 123, P 0187). Row 11: Dietary beans, < 3/week (reference), >= 3/week (AOR 083, 95% CI 044, 155, P 0557). Row 12: Dietary milk, < 3/week (reference), >= 3/week (AOR 143, 95% CI 072, 281, P 0306). Row 13: Dietary meats, < 3/week (reference), >= 3/week (AOR 134, 95% CI 064, 279, P 0442). Row 14: Dietary eggs, < 3/week (reference), >= 3/week (AOR 084, 95% CI 042, 168, P 0617). Row 15: Sugary drink, < 3/week (reference), >= 3/week (AOR 066, 95% CI 029, 154, P 0339). Row 16: Dietary vegetables, < 3/week (reference), >= 3/week (AOR 049, 95% CI 010, 255, P 0400). Row 17: Dietary fruits, < 3/week (reference), >= 3/week (AOR 086, 95% CI 044, 168, P 0656). Row 18: Dietary nuts, < 3/week (reference), >= 3/week (AOR 141, 95% CI 064, 311, P 0390).

Navigate back to Table 5.

## Table 6 Long description

The table presents a multivariate Poisson regression analysis of factors associated with overnutrition and undernutrition-related disorders among school-age children and adolescents in Western China. It has 12 rows and 6 columns. The columns are labeled Characteristics, PR, 95 % CI, and P values for both overnutrition-related disorders and undernutrition-related disorders. The rows are labeled with various characteristics such as sex, age group, weight group, physical activity, screen time, age of mother, age of father, education of mother, education of father, and household size. Each row provides the prevalence ratio (PR), 95 percent confidence interval (CI), and P values for the association of each characteristic with overnutrition and undernutrition-related disorders. Notable trends include older age groups, overweight, and obesity being positively associated with overnutrition-related disorders, while higher physical activity and larger household size are negatively associated. For undernutrition-related disorders, being female and having higher screen time are positively associated, while higher education levels of the father are negatively associated.

Navigate back to Table 6.
